# Lymphangiome du sein: à propos d’un cas

**DOI:** 10.11604/pamj.2017.28.23.12318

**Published:** 2017-09-13

**Authors:** Eric Mbuya Musapudi, Aimé Lukoba Bwalya, Igor Mujinga wa Mujinga, Didier Tshibangu Mujinga, Julien Ilunga Nikulu, Guy Nday Ilunga

**Affiliations:** 1 Département de Chirurgie, Cliniques Universitaires de Lubumbashi, Faculté de Médecine, Université de Lubumbashi, Lubumbashi, Republique Démocratique du Congo; 2 Pathologiste Cliniques Universitaires de Lubumbashi, Faculté de Médecine, Université de Lubumbashi, Lubumbashi, Republique Démocratique du Congo

**Keywords:** tumeur bénigne du sein, lymphangiome, vaisseaux lymphatiques, prise en charge chirurgicale, Benign tumor of the breast, lymphangioma, lymphatic vessels, surgical management

## Abstract

Le lymphangiome est une lésion bénigne de vaisseaux lymphatiques. C'est une affection pédiatrique rare et exceptionnelle chez l'adulte. Le site habituel est le cou, l'aisselle et l'abdomen. La localisation mammaire est exceptionnelle. Nous rapportons l'observation d'une patiente âgée de 18 ans reçue en consultation pour une masse du sein gauche évoluant depuis 23 mois. La prise en charge était chirurgicale par une exérèse complète. L'examen anatomo-pathologique a conclu à un lymphangiome du sein. Son évolution a été bonne et sans récidive après 10 mois de suivi.

## Abstract (en)

Lymphangioma is a benign lesion of the lymphatic vessels. It is a rare paediatric condition and it is exceptional among adults. It mainly occurs in the neck, armpit and abdomen. It rarely occurs in the breast. We report the case of a 18 year-old female patient seen in consultation for a mass in her left breast, which had evolved over the last 23 months. Management was based on surgery. The patient underwent complete tumor resection. Anatomopathological examination showed lymphangioma of the breast. Patient’s evolution was favorable and the patient had a median 10 months follow-up with no recurrences identified.

## Introduction

Le lymphangiome est une tumeur bénigne des vaisseaux lymphatiques. Il semble être d'origine congénitale chez l'enfant, alors que les lymphangiomes de l'adulte suivent le plus souvent une obstruction lymphatique secondaire à une pathologie extra-lymphatique [1]. Les lymphangiomes sont classés en trois types, capillaires, caverneux et kystiques. La distinction entre lymphangiome kystique et lymphangiome caverneux est souvent arbitraire, du fait de la présence souvent des deux composantes dans la même lésion [2]. Le lymphangiome kystique est une affection rare, il représente 5% des anomalies vasculaires. Son incidence annuelle est comprise entre 1 et 8 pour 100000 hospitalisations. Il est fréquent chez les enfants de moins de 2 ans (90%) et rare chez l'adulte [3]. Le diagnostic est anatomopathologique. La prise en charge curative du lymphangiome est chirurgicale, par une exérèse complète, par contre, la récidive peut être notée lorsqu'il est incomplètement reséqué [4]. Le site le plus habituel est le cou (70%), l'aisselle (20%) et l'abdomen (10%) [3]. Elle est due à une dilatation des canaux lymphatiques bordés par l'endothélium [2]. La localisation mammaire est exceptionnelle. En 2010, moins de 20 cas de lymphangiomes mammaires étaient rapportés dans la littérature, avec un seul cas chez une femme africaine, signale une revue marocaine du cancer [2]. La rareté de cette pathologie de part sa fréquence et surtout sa localisation, motive la présente publication. L'objectif a été de décrire un cas de lymphangiome mammaire diagnostiqué et prise en charge aux cliniques universitaires de Lubumbashi, Katanga, RDC, Mai 2016.

## Patient et observation

**Figure 1:**
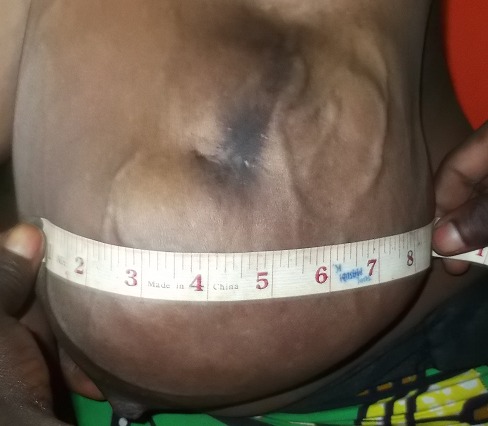
Aspect clinique de la tumeur du sein gauche occupant le quadrant supéroexterne avec une circulation collatérale, le mamelon est refoulé en bas

**Figure 2:**
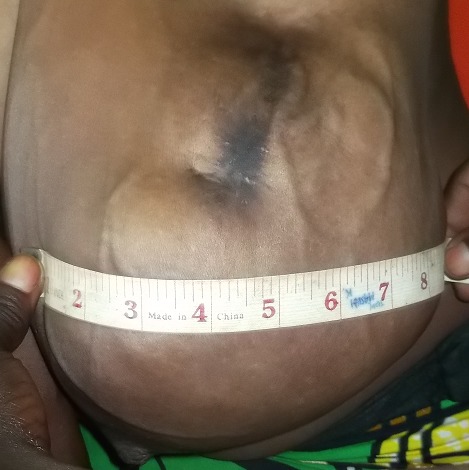
Aspect de la tumeur du sein, présence d’une cicatrice à son pôle supérieur (survenue après application de traitement traditionnel)

**Figure 3:**
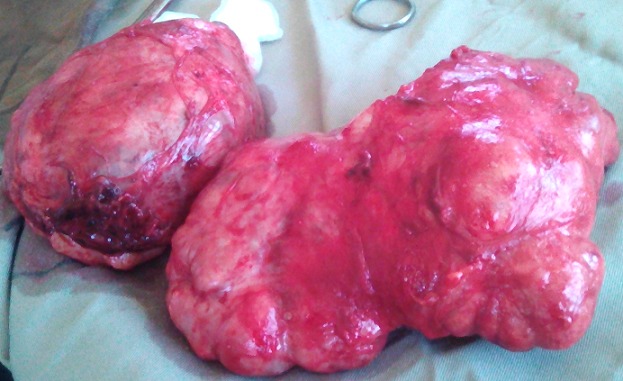
Exposition des tumeurs après leur exérèse chirurgicale

Il s'agissait d'une patiente âgée de 18 ans, reçue en consultation dans le service de chirurgie des cliniques universitaires de Lubumbashi en date du 11 Mai 2016. Le motif de consultation était une masse du sein gauche évoluant depuis 23 mois. Le traitement traditionnel reçu auparavant avait occasionné l'apparition d'une solution de continuité et une suppuration au niveau du sein. Aucune notion de traitement hormonal substitutif n'avait été retrouvée aux antécédents. Elle avait vu ses ménarches à l'âge de 13 ans. Elle était mère de 2 enfants dont le dernier avait 7 mois d'âge et nourri au lait maternel. Elle avait constaté cette masse alors qu'elle portait la grossesse de son premier enfant. L'examen physique a montré, une masse localisée dans les deux quadrants externes supérieur et inférieur du sein gauche (Figure 1), d'environ 12 cm de grand axe. La peau en regard était saine, avec une circulation collatérale. Une cicatrice hyperpigmentée a été objectivée au pôle supérieur de la masse (lésion d'application de produits indigènes) (Figure 2). Le mamelon était refoulé vers le bas par la masse. La chaleur locale était conservée. La masse était ferme, multinodulaire, indolore, n'adhérant ni au plan profond ni au plan superficiel. Il y avait la présence d'une adénopathie axillaire d'environ 2 cm de grand axe, ferme, douloureuse avec périadénite. La mammographie et IRM n'ont pas été réalisées. La radiographie du thorax face n'avait rien révélé de particuliers. Après un bilan préopératoire réalisé, dont, le groupe sanguin (A rhésus positif), Hb (12.2g%), Hct (36%), temps de saignement (3 minutes et 0 seconde), temps de coagulation (6 minutes et 0 seconde) et une visite pré-anesthésique, la tumorectomie a été programmée. L'intervention chirurgicale réalisée 2 jours après son admission, a permis l'exérèse complète de deux masses multinodulaires (Figure 3) favorisée par un plan de clivage bien net. L'analyse anatomopathologique de ces 2 masses extirpées avait montré, macroscopiquement, qu'elles étaient multinodulaires mesurant respectivement, 16x7x5cm et 16x12x8cm. La microscopie a révélé, un parenchyme mammaire d'architecture partiellement conservée, la présence des structures cavitaires et une importante réaction fibreuse. Les structures cavitaires étaient bordées par un épithélium de type lymphatique. L'existence d'un infiltrat inflammatoire dans le tissu fibro-conjoctif était observée. Le diagnostic d'un lymphangiome caverneux à localisation mammaire était ainsi retenu. Les suites post-opératoires étaient simples. Les fils de suture cutanée ont été enlevés au 10^ème^ jour et la patiente était sortie de l'hôpital au 12^ème^ jour. Aucun traitement adjuvant ne lui a été administré. Elle est actuellement au 10^ème^ mois post-tumorectomie sans signes de récidive avec des seins symétriques.

## Discussion

Deux hypothèses expliquent la physiopathologie de lymphangiome. La première explique les lymphangiomes hamartomateux comme des malformations qui résultent de l'échec du système lymphatique à communiquer avec le système veineux. La deuxième hypothèse évoquée, c'est une séquestration du tissu lymphatique mammaire qui ne parvient pas à communiquer normalement avec le système lymphatique. Ce dernier possède une certaine capacité à se multiplier et à accumuler de grandes quantités de liquide, à l'origine de l'aspect kystique [2]. Il existe plusieurs classifications des lymphangiomes. La classification actuelle les divise en lésions micro kystiques (composées d'éléments inférieurs à 2cm³), macro kystiques (espaces kystiques de volume supérieur à 2 cm³) et mixtes (qui contiennent les deux types de kyste) [5]. D'autres auteurs préfèrent diviser ces malformations en lymphangiomes capillaires, caverneux et kystiques mais les limites entre les groupes ne sont pas toujours nettes [3]. Dans notre cas il s'agissait du type caverneux. L'âge de diagnostic varie selon les cas. Benna en Tunisie [6] décrit un cas d'un lymphangiome mammaire chez une patiente de 54 ans, Boufettal au Maroc [2], chez celle de 69 ans tandis que, Hiremath [7] et Ravikanth [3] en inde décrivent deux cas différents avec 23 ans respectivement. Dans notre cas, il s'agit d'un adulte de 18 ans. Les lymphangiomes sont le plus souvent, des lésions bénignes à croissance lente qui ne se transforment pas en tumeur maligne [8]. Ces tumeurs sont fréquemment localisées au niveau de la tête, du cou, dans l´aisselle et en intra-abdominale [9]. La localisation mammaire retrouvée chez notre patiente, est exceptionnelle. Et ceci rejoint les avis des autres auteurs. [2, 3, 6,7] Ils sont habituellement asymptomatiques et se développent très lentement [3]. La douleur et l´inconfort peuvent être vécus à mesure qu´ils augmentent de taille, comme les lymphangiomes observés chez les femmes enceintes ou allaitantes [6]. Plusieurs auteurs [3, 4,8] retrouvent la tuméfaction douloureuse comme principal motif de consultation. L'augmentation du volume de la masse chez notre patiente qui était allaitante était le motif principal de consultation. La plupart de lymphangiomes du sein qui sont décrits dans la littérature se localisent au niveau du quadrant supéroexterne [4,8], rarement au quadrant antéroexterne comme celui retrouvé par Benna en Tunisie [6].

Les travaux anatomiques de Sappey montrent que tous les lymphatiques du sein se dirigent vers le mamelon en constituant un plexus sous-aréolaire. C'est à partir de ce plexus que se constituent deux canaux lymphatiques principaux qui se dirigent directement ou indirectement vers l'aisselle [10]. Ceci explique pourquoi, la plupart de lymphangiomes se localisent au quadrant supéroexterne. Chez notre patiente, la tumeur occupait les deux quadrants externes supérieur et inferieur. L'adénopathie axillaire qui a été mise en évidence chez notre patiente avait un caractère inflammatoire. Elle pouvait être liée aux différentes complications de l'allaitement notamment, les crevasses du mamelon négligées qui peuvent être à l´origine d´un engorgement unilatéral, puis d´une lymphangite. Elle pourrait être aussi être secondaire à une surinfection de la plaie qui avait fait suite au traitement traditionnel appliqué sur la tumeur. Cette adénopathie avait disparu à la dernière consultation soit au 6eme mois après l'intervention. L´évaluation radiologique d´une masse palpable comprend plusieurs modalités. La mammographie montre généralement une lésion lobulée ou ronde avec une densité tissulaire accrue. L´échographie standard permet de différencier les masses solides des masses kystiques. Actuellement, l'imagerie par résonnance magnétique est considérée comme le meilleur examen pour diagnostiquer les lymphangiomes [8]. Ces examens d'imagerie n'ont pas été réalisés dans notre cas. Du point de vu macroscopique, le lymphangiome peut être macronodulaire ou micronodulaire [9]. Dans notre cas, il était macronodulaire. Comme l'a constaté Ravikanth en Inde [3]. La confirmation du diagnostic était histopathologie montrant la présence des structures cavitaires et d'une importante réaction fibreuse. Les structures cavitaires étaient bordées par un épithélium de type lymphatique. On note en outre l'existence d'un infiltrat inflammatoire dans le tissu fibro-conjonctif. Nous avons retenu la forme de lymphangiome caverneux pour le cas présenté, comme cela a été constaté dans d'autres observations [4, 7]. L'exérèse chirurgicale complète de la tumeur représente la meilleure modalité de traitement de cette maladie bénigne du sein [2]. Cependant, la récidive peut être observée lorsque l'exérèse est incomplète [4]. Dans notre cas, le traitement était chirurgical par une exérèse de la tumeur et nous n'avons pas noté de récidive 10 mois après l'intervention chirurgicale.

## Conclusion

Le lymphangiome est une tumeur bénigne rare de vaisseaux lymphatiques. Sa localisation mammaire est exceptionnelle. Son diagnostic est anatomopathologique et son traitement curatif consiste en une exérèse chirurgicale complète de la tumeur.

## Conflits d’intérêts

Les auteurs ne déclarent aucun conflit d'intérêts.
